# Stat3-mediated alterations in lysosomal membrane protein composition

**DOI:** 10.1074/jbc.RA118.001777

**Published:** 2018-01-17

**Authors:** Bethan Lloyd-Lewis, Caroline C. Krueger, Timothy J. Sargeant, Michael E. D'Angelo, Michael J. Deery, Renata Feret, Julie A. Howard, Kathryn S. Lilley, Christine J. Watson

**Affiliations:** From the ‡ Department of Pathology, University of Cambridge, Cambridge CB2 1QP, United Kingdom,; the § Lysosomal Diseases Research Unit, South Australian Health and Medical Research Institute, Adelaide, South Australia 5000, Australia, and; the ¶ Cambridge Centre for Proteomics, Department of Biochemistry, University of Cambridge, Tennis Court Road, Cambridge CB2 1QR, United Kingdom

**Keywords:** mammary gland, STAT3, cell death, lysosome, proteomics, involution, lysosome purification, mammary epithelial cells

## Abstract

Lysosome function is essential in cellular homeostasis. In addition to its recycling role, the lysosome has recently been recognized as a cellular signaling hub. We have shown in mammary epithelial cells, both *in vivo* and *in vitro*, that signal transducer and activator of transcription 3 (Stat3) modulates lysosome biogenesis and can promote the release of lysosomal proteases that culminates in cell death. To further investigate the impact of Stat3 on lysosomal function, we conducted a proteomic screen of changes in lysosomal membrane protein components induced by Stat3 using an iron nanoparticle enrichment strategy. Our results show that Stat3 activation not only elevates the levels of known membrane proteins but results in the appearance of unexpected factors, including cell surface proteins such as annexins and flotillins. These data suggest that Stat3 may coordinately regulate endocytosis, intracellular trafficking, and lysosome biogenesis to drive lysosome-mediated cell death in mammary epithelial cells. The methodologies described in this study also provide significant improvements to current techniques used for the purification and analysis of the lysosomal proteome.

## Introduction

Lysosomes are intracellular organelles, so-named from the Greek words lysis (dissolution or destruction) and soma (body), that were first described by Christian de Duve over half a century ago (1). The lysosome has an essential function in the digestion of old organelles, engulfed proteins, and microbes that are delivered to the lysosome for degradation by autophagy, endocytosis, and phagocytosis, respectively. Delivery is mediated by kissing events between lysosomes and late endosomes/multivesicular bodies or by direct fusion of lysosomes and autophagosomes (2). Lysosomes can also fuse with the plasma membrane to mediate repair and exocytosis (3). Suboptimal lysosome function and reduced lysosomal clearance is associated with aging, neurodegenerative diseases such as Alzheimer's, and lysosomal storage disorders caused by deficiencies in lysosomal proteins or trafficking (4). The lysosomal lumen harbors numerous acid hydrolases that digest a wide array of cellular macromolecules and membranes with the resulting catabolites being recycled back to the cytosol (5). Lysosomal proteases have multiple additional roles, including bone remodeling, angiogenesis, neuronal cell maintenance, cell death, and cancer cell invasion (6).

The lysosomal proteome has been investigated using a variety of mass-spectrometry (MS)–based approaches, including affinity purification methods that exploit the mannose 6-phosphate modifications required to traffic proteins to the lysosome (7–11). Such approaches have enabled the identification of both known and previously uncharacterized proteins involved in lysosome biology (8). However, sorting signals present on lysosomal membrane proteins are less amenable to purification, and difficulties in isolating pure populations of lysosomes have hindered proteomic analysis of lysosomal membrane constituents. As a result, this proteome is considered relatively incomplete (12). Traditional methods for isolating lysosomes have relied upon density centrifugation, resulting in preparations that are commonly contaminated by other organelles of similar density, including mitochondria and peroxisomes (8, 13–15). Protein correlation profiling can partially mitigate this problem and provide information about the steady-state distribution of proteins within organelles (16, 17). However, lysosomes can be highly heterogeneous in nature, and in biological contexts where their function is impaired or altered (*e.g.* in lysosomal storage disorders) changes in lysosomal buoyant density result in their redistribution across a density gradient, thus hampering their purification by differential centrifugation (13, 18, 19). To overcome these difficulties, we and others have utilized magnetic iron nanoparticles to isolate lysosomes from different cell types (13, 18, 20, 21). The internalization and delivery of nanoparticles via the endocytic pathway to lysosomes enables their isolation by magnetic chromatography. More recently, the utility of this method for LC-MS/MS analysis of cellular trafficking events has been demonstrated (22, 23). However, the application of this methodology to specifically investigate changes in the lysosomal membrane proteome in the context of lysosomal dysfunction (including during lysosomal membrane permeabilization (LMP))^4^ has yet to be explored.

Components of the lysosomal membrane fulfil a number of crucial functions, including acidification of the lysosomal lumen, membrane fusion with other organelles, and transport events that facilitate the transfer of macromolecules and degradation products (12, 24). Preservation of lysosomal function requires the multicomponent vacuolar-type ATPase to maintain the acidic luminal pH. The role of the densely glycosylated proteins LAMP1 and LAMP2, which constitute over 50% of the lysosomal membrane proteins (12), is less clear. It has been suggested that they form a glycocalyx that protects the lysosomal membrane from autodigestion (12, 25). However, other studies indicate that they are not required simply for membrane stability (12), although glycosylation is necessary to protect LAMP1/2 from the action of lysosomal proteases (25). LMP, and the subsequent intracellular release of lysosomal hydrolases such as cathepsin proteases, is widely implicated in the initiation or enhancement of cell death programs (26). Although cathepsin release may result in the activation of the caspase cascade, cell death can also be initiated in a caspase-independent manner (27) in a process termed lysosome-mediated programmed cell death (28). The mechanisms driving LMP appear to be highly cell-type– and context–dependent and have been observed across a wide spectrum of species including *Caenorhabditis elegans* (29). In addition to mediating cell death in pathological conditions, lysosome-mediated programmed cell death can regulate cell death under physiological conditions, such as during post-lactational regression (involution) of the mammary gland (20, 30). This complex and highly regulated program of cell death requires Stat3 signaling that coordinately up-regulates the lysosomal system and abrogates expression of the endogenous cathepsin inhibitor Spi2a (30, 31). Subsequent LMP and leakage of cathepsin proteases into the cytosol results in extensive cell death (30). More recently, we have shown that Stat3 activation mediates the uptake of milk-fat globules that are delivered to large lysosomal vesicles for degradation (20). The resulting high local concentrations of free fatty acids within these structures lead to increased membrane permeability, cathepsin leakage, and cell death (20). These events can be modeled *in vitro* using oncostatin M (OSM) stimulation of Stat3 activity in the normal mouse mammary epithelial EpH4 cell line (20, 30). It is unclear, however, whether Stat3 signaling has a direct, modulatory effect on the lysosomal membrane proteome. Here, we utilized LC-MS/MS analysis of lysosomes isolated from OSM-stimulated or unstimulated EpH4 cells to address this question and to provide further insights into the protein composition of lysosomal membranes during lysosome-mediated programmed cell death.

## Results

### Iron nanoparticles facilitate the isolation of highly pure lysosomal preparations from EpH4 cells for mass-spectrometry analysis

**Figure 1.**
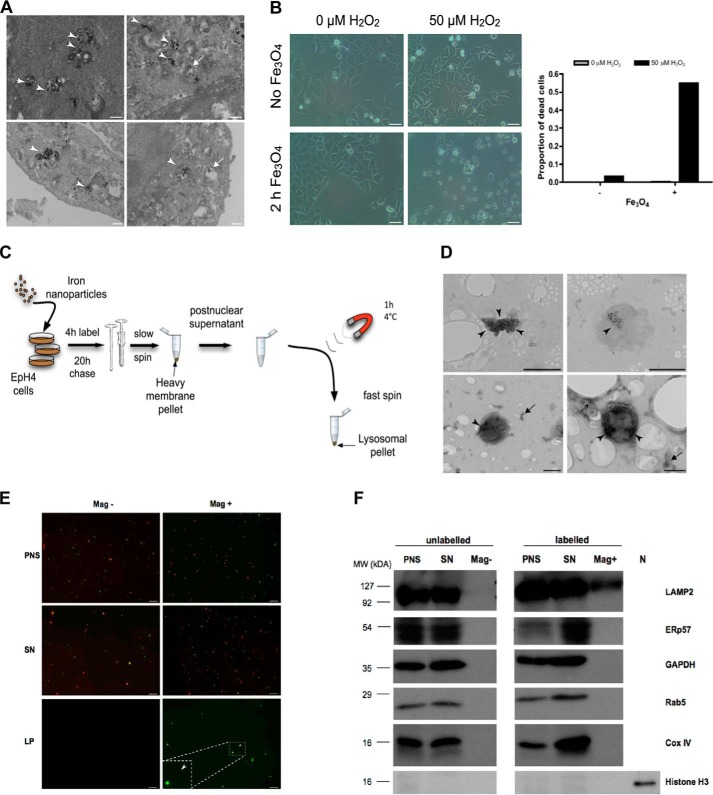
**Iron nanoparticle-mediated isolation of EpH4 cell lysosomes.** *A*, TEM images of EpH4 cells showing iron nanoparticles residing in degradative lysosomal vacuoles (*arrowheads*) or in lysosomal vacuoles devoid of degradative material (*arrows*). *Scale bars*: 500 nm. *B*, bright-field microscopy and quantification of iron nanoparticle–induced cell death of EpH4 cells observed solely in the presence of hydrogen peroxide (H_2_O_2_). EpH4 cells were treated with H_2_O_2_ for 19 h. *Scale bars*: 25 μm. *C*, schematic of the magnetic iron nanoparticle lysosomal purification protocol. Adapted from Sargeant *et al.* (20). *D*, TEM images of lysosomes (*top panel*) and negatively stained lysosomes (*bottom panel*) isolated using the magnetic iron nanoparticle purification protocol. *Arrowheads* mark areas containing iron nanoparticles. *Arrows* indicate unidentifiable membranous fragments, which may be endolysosomal tubules or remnants from the ER or Golgi apparatus. *Scale bars*: 500 nm. *E*, isolated EpH4 lysosomes are predominantly clear of mitochondria. EpH4 cells were labeled with 10 kDa FITC-dextran (2.5 mg/ml (*green*)) with (*Mag*+) or without (*Mag*−) iron nanoparticle–containing media for 2 h and subsequently stained with MitoTracker^TM^ (500 nm (*red*)) for 30 min prior to isolation. Representative images of the PNS, post-magnetic SN, and magnetic lysosomal pellet (*LP*) of the two conditions are shown. *Scale bar*: 10 μm. *F*, isolated EpH4 lysosomes are highly pure with undetectable contamination from other organelles as observed by immunoblotting. *N*, nuclear lysate. Organelle marker proteins: LAMP2, lysosomes; ERp57, endoplasmic reticulum; GAPDH, cytoplasm; Rab5, early endosomes; Cox IV, mitochondria; Histone H3, nucleus.

Previously, we developed a magnetic iron nanoparticle protocol to isolate functional lysosomes for membrane permeability studies(20). To investigate the suitability of these preparations for downstream MS analysis, we sought to further characterize the lysosomes isolated using this method. By transmission electron microscopy (TEM) we observed that fluid phase uptake of nanoparticles by EpH4 cells led to the specific loading of degradative lysosomal vacuoles (Fig. 1*A*). Importantly, these particles were nontoxic, and cytotoxicity was observed only in the presence of hydrogen peroxide (Fig. 1*B*), likely a consequence of reactive free radicals generated by the Fenton reaction (32). Using iron nanoparticles, we were able to enrich and isolate EpH4 lysosomes with relative ease (Fig. 1*C*). The lysosomal identity of the isolated organelles was confirmed by TEM analysis, which showed the presence of vacuolar structures containing magnetic nanoparticles (Fig. 1*D*, *upper panel*). Furthermore, negative staining of samples also revealed the presence of iron-containing membrane-bound organelles having a size and morphology consistent with lysosomes (Fig. 1*D*, *lower panel*). Importantly, fluorescence imaging and Western blot analysis of iron-labeled (Mag^+^) preparations revealed nearly undetectable levels of mitochondrial proteins (Fig. 1, *E* and *F*), a common contaminant of lysosomal preparations isolated using methods such as density gradient centrifugation. In addition, contamination by other organelles, including endocytic vesicles and endoplasmic reticulum (ER), was minimal (Fig. 1*F*), demonstrating the effectiveness of this method for yielding highly pure lysosomal preparations.

**Figure 2.**
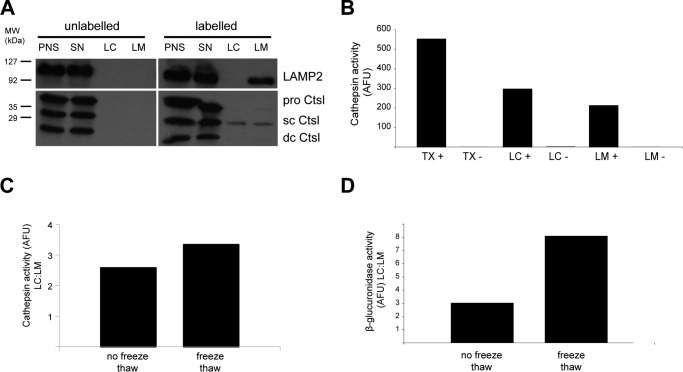
**Membrane fractionation of isolated EpH4 lysosomes.** *A*, hypotonic lysis of lysosomes isolated from EpH4 cells using iron nanoparticles to separate the LM from the LC. LAMP2 immunoblotting shows its enrichment in the LM fraction. *Ctsl*, cathepsin L (*sc*, single chain: *dc*, double chain); *SN*, post-magnetic supernatant. *B*, cathepsin activity in EpH4 lysosomes isolated using iron nanoparticles and extracted using TX-100 (*TX*+, total lysosomal content) or by hypotonic lysis and fractionation (*LM*+, membrane fractions; *LC*+, matrix) compared with unlabeled control samples (*TX*−, *LM*−, and *LC*−). *C* and *D*, freeze-thawing of lysosomes improved lysosome membrane separation from the lysosomal matrix. Cathepsin (*C*) and β-glucuronidase activity (*D*) in the LC fraction is shown as -fold of LM. *AFU*, arbitrary fluorescence units.

To further enrich for lysosomal membrane proteins and to reduce the contribution from cargo delivered to the lysosomes for degradation, isolated preparations were subjected to hypotonic lysis and centrifugation to separate lysosomal membranes. Using this method, the lysosomal membrane protein LAMP2 could be specifically detected in lysosomal membrane (LM) fractions (Fig. 2*A*). However, the lysosomal hydrolase cathepsin L (*Ctsl*) was detected at comparable levels in both the LM and the soluble compartments (LC, lysosomal content) of purified lysosomes (Fig. 2, *A* and *B*), suggesting that not all organelles were successfully ruptured. To overcome this potential issue, preparations were subsequently subjected to repeated cycles of freeze-thawing. This optimization improved the enrichment of cathepsin activity in the soluble fraction (Fig. 2*C*), indicating the enhanced disruption of lysosomes. We also assessed the activity of β-glucuronidase, a lysosomal enzyme that is predominately localized in the lysosomal matrix and has minimal adherence to membranes (33), to more definitively assess the degree of lysosomal membrane disruption. As expected, β-glucuronidase activity in the soluble fraction was higher than in the membrane preparations and was markedly increased by freeze-thawing (Fig. 2*D*). Therefore, hypotonic lysis of isolated lysosomes, combined with freeze fracture, facilitated the enrichment of lysosomal membranes for downstream MS analysis.

### Mass-spectrometry analysis of EpH4 cell lysosomal membrane preparations isolated using iron nanoparticles

To further validate and assess the efficacy of the optimized lysosomal membrane isolation procedure prior to undertaking large-scale experiments, a pilot preparation was submitted for LC-MS/MS analysis. Here, 1664 proteins were identified by MS (Table S1*A*), which notably included numerous known lysosomal membrane proteins (8, 34) (Table 1), thus validating the utility of our lysosomal purification method for downstream proteomic analysis. Proteins that were also identified in the corresponding unlabeled (no magnetic particles) control sample (327 proteins, Table S1*B*) and that did not belong to the endocytic-lysosomal pathway were removed from this list. Furthermore, published common contaminants of MS experiments (35), such as heat-shock proteins, keratins, tubulins, actins, elongation factors, histones, and ribosomal and ribonucleoproteins, were also removed. This resulted in a subset of 1295 proteins (Table S2). To obtain a functional overview of the proteins identified, the Kyoto Encyclopedia of Genes and Genomes (KEGG) pathway and gene ontology (GO) enrichment analysis was undertaken using the online gene list enrichment analysis tool Enrichr (36, 37) (http://amp.pharm.mssm.edu/Enrichr/).^5^ Analysis of the KEGG pathway annotations associated with this subset revealed a significant enrichment in the proteins involved in the lysosomal and related pathways, including neurodegenerative diseases allied with lysosomal dysfunction (Fig. S1*A* and Table S3*A*). Furthermore, GO term distribution analysis for the cellular component confirmed the lysosomal and vesicular membrane localization of the identified proteins (Fig. S1*B* and Table S3*B*). Functional analysis also revealed the enrichment of mitochondrial membrane proteins involved in oxidative phosphorylation (Fig. S1*A* and *B*). This suggests that lysosomal preparations isolated using iron nanoparticles may contain a degree of mitochondrial contamination that is undetectable by Western blotting or MitoTracker^TM^ analysis (*e.g.* Fig. 1, *E* and *F*). However, it is important to note that this contamination may also reflect the delivery of damaged mitochondria to the lysosome for turnover during mitophagy (20, 38) or the remnants of lysosomal-mitochondrial contact sites (39, 40).

**Table 1:** **Known lysosomal membrane proteins identified in lysosomal preparations isolated using iron nanoparticles** Published lysosomal membrane proteins that were identified by mass spectrometry in a preliminary experiment to validate the iron nanoparticle–mediated lysosomal isolation protocol (associated with Table S1).

Protein	Reference	Protein	Reference
H^+^-ATPase V_1_ subunit A	(34)	LIMP2	(66)
H^+^-ATPase V_1_ subunit B2	(34)	Sialin	(67)
H^+^-ATPase V_o_ 116-kDa subunit a isoform 1	(34)	CLC-7	(68)
H^+^-ATPase V_o_ subunit d	(34)	ATPase 13A2	(69)
H^+^-ATPase V_1_ 56/58-kDa subunit B1	(34)	Mucolipin-1	(70)
H^+^-ATPase V_1_ subunit H splice isoform 1	(34)	rPHT2	(71)
H^+^-ATPase V_1_ subunit C	(34)	GLUT-8	(72)
H^+^-ATPase V_1_ subunit E	(34)	Equilibrative nucleoside transporter 3	(73)
H^+^-ATPase V_o_ 116-kDa subunit a isoform 2	(34)	Natural resistance-associated macrophage protein 2	(74)
H^+^-ATPase V_1_ subunit D	(34)	Zinc transporter 2	(75)
H^+^-ATPase V_1_ subunit G1	(34)	ATP-binding cassette subfamily A member 3	(76)
S1 accessory proteins (Ac45)	(34)	ATP-binding cassette subfamily B member 9	(77)
H^+^-ATPase V_o_ 16-kDa proteolipid subunit c	(34)	Probable lysosomal cobalamin transport	(78)
Glucosylceramidase	(34)	Major facilitator superfamily domain--containing protein 8	(79)
Arf-like 10C (Arl8B)	(34)	Heparan-α-glucosaminide *N*-acetyltransferase	(80)
Flotillin 1	(34)	Signal peptide peptidase-like 2A	(81)
Arf-like 10B (Arl8A)	(34)	Nicastrin	(82)
Lysosomal α-glucosidase	(34)	Presenilin1	(82)
SLC29A3 nucleoside transporter	(34)	Transmembrane protein 55A	(83)
Chloride channel 5, CLC5	(34)	Transmembrane protein 55B	(83)
MLN64 N-terminal domain homolog	(34)	Vesicle-associated membrane protein 7	(84)
Phospholipase D1 isoform, PLD1A	(34)	Transmembrane protein 192	(34)
Endothelin-converting enzyme 1 isoform B	(34)	Lysosomal protein NCU-G1	(85)
C18orf8 protein, Mic1	(34)	Transmembrane protein 63A	(8)
SID1 transmembrane protein, SIDT2	(34)	Niemann-Pick C1 protein	(86)
GPR137 protein	(34)	Niemann-Pick C2 protein	(87)
LAMP1	(88)	Osteopetrosis-associated transmembrane protein 1	(68)
LAMP2	(88)		
CD63/LIMP1	(89)		

### Oncostatin M stimulates vesicle biogenesis in EpH4 cells

**Figure 3.**
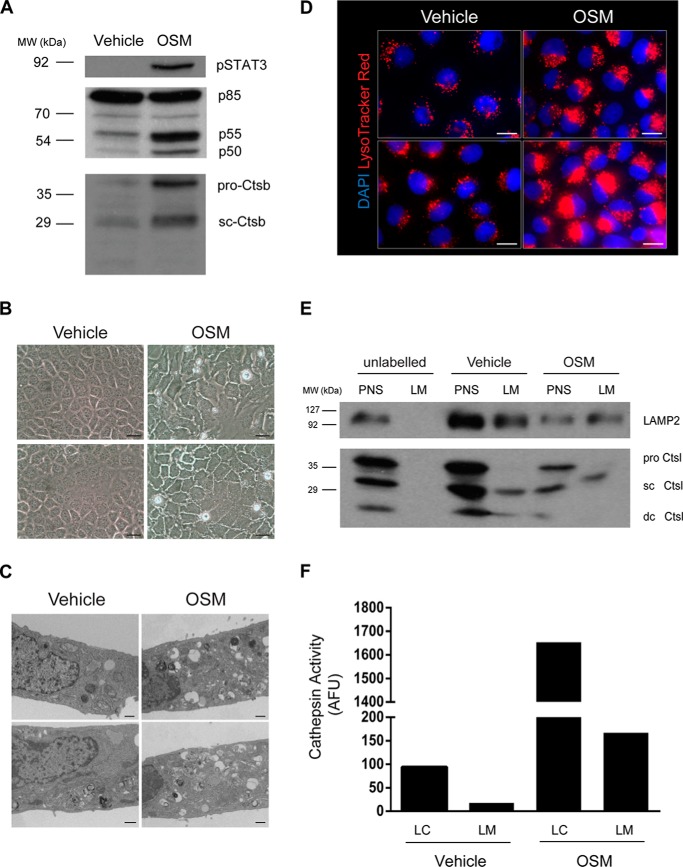
**OSM induces vesicular biogenesis in EpH4 cells.** *A*, immunoblot showing OSM-induced expression of p55α/p50α and cathepsin B (*Ctsb*; *sc*, single chain) downstream of activated Stat3 signaling in EpH4 cells. *B*, bright-field microscopy showing OSM-induced vacuolization in EpH4 cells treated for 72 h. *Scale bars*: 25 μm. *C*, TEM images showing an increased number of degradative vesicles after 72 h of OSM stimulation. *Scale bars*: 500 nm. *D*, fluorescence microscopy of LysoTracker® accumulation in cells treated with OSM for 72 h. *Scale bars*: 20 μm. *E*, Western blot analysis of LAMP2 and cathepsin L in fractionated lysosomes isolated from vehicle and OSM stimulated (72 h) cells using iron nanoparticles. *sc Ctsl*, single-chain cathepsin L; *dc Ctsl*, heavy chain of the double-chain form of cathepsin L. *F*, graph showing increased cathepsin activity in LC and LM fractions from OSM-stimulated cells. *AFU*, arbitrary fluorescence units.

We have shown previously that stimulation of EpH4 mammary epithelial cells by OSM leads to LMP (30). OSM-induced Stat3 phosphorylation in EpH4 cells resulted in the increased expression of multiple target genes, including the phosphatidylinositol 3-kinase subunits p55α and p50α (41) and the lysosomal protease cathepsin B (Fig. 3*A*, *Ctsb*), alongside increased vacuole formation (Fig. 3*B*) (20), which appeared to be degradative in nature (Fig. 3*C*). These structures accumulate the lysosomotropic dye LysoTracker® Red, thus revealing their lysosomal origin (Fig. 3*D*). Notably, the apparent size of the lysosomal compartment is increased by OSM treatment and is associated with a more perinuclear localization (Fig. 3*D*).

To further characterize Stat3-induced changes to the lysosomal compartment, we sought to isolate lysosomes from OSM-stimulated EpH4 cells using the optimized magnetic nanoparticle fractionation protocol. Western blot analysis of the lysosomal markers LAMP2 and cathepsin L demonstrated that this technique was also applicable to OSM-treated cells and that the stimulation did not significantly interfere with iron uptake (Fig. 3*E*). Interestingly, OSM treatment resulted in increased LAMP2 glycosylation, as observed by the molecular weight shift in LAMP2 on Western blot analysis (Fig. 3*E*). Prolonged OSM treatment resulted in cell death (Fig. 3*B*) (30), leading to a reduction in protein yields as compared with control-treated EpH4 cells (Fig. 3*E*, *PNS lane*). Nevertheless, OSM-mediated induction of cathepsin L expression corresponded to an approximately 16-fold increase in enzyme activity inside the lysosomal matrix (Fig. 3*F*, *LC*).

### OSM-induced changes to the EpH4 lysosomal proteome

**Figure 4.**
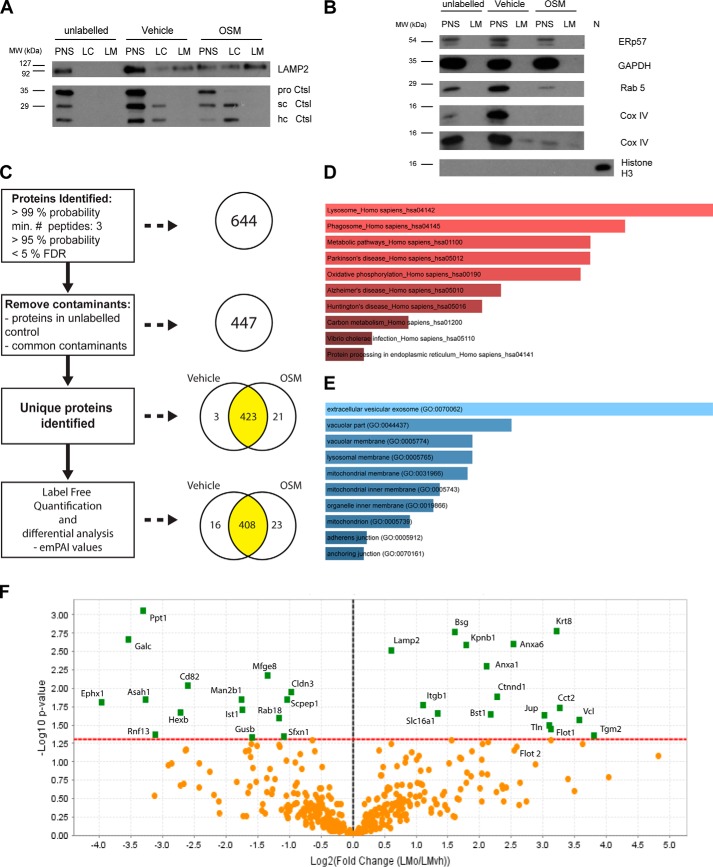
**OSM-induced changes in the lysosomal proteome of EpH4 cells.** *A*, Western blot analysis of LAMP2 and cathepsin L in fractionated lysosomes isolated from vehicle and OSM-stimulated (72 h) cells and submitted for MS analysis. A representative blot from three independent experiments is presented. *sc Ctsl*, single-chain cathepsin L; *hc Ctsl*, heavy chain of the double-chain form of cathepsin L. *B*, immunoblotting for organelle marker proteins in lysosomal preparations isolated from control and OSM-stimulated EpH4 cells: ERp57, endoplasmic reticulum; GAPDH, cytoplasm; Rab5, early endosomes; Cox IV, mitochondria; Histone H3, nucleus. A representative blot from three independent experiments is presented. For Cox IV immunoblotting, blots from two different replicates are shown, revealing slight contamination in one replicate. *C*, analysis pipeline of mass spectrometry results obtained from lysosomes isolated from vehicle and OSM (72 h)-treated EpH4 cells. Three independent biological repeats were undertaken per condition, and each replicate included an unlabeled control samples (nine preparations in total). *D*, significantly enriched KEGG (37) pathways derived from MS analysis of EpH4 lysosomal preparations isolated using iron nanoparticles. *E*, significant GO annotations of proteins according to cellular component, derived from mass-spectrometry analysis of EpH4 lysosomal preparations isolated using iron nanoparticles. For inclusion in the analysis undertaken in *D* and *E*, proteins were required to be identified in at least five of six independent runs (3 vehicle, 3 OSM). *F*, volcano plot showing the -fold change in emPAI values induced by OSM stimulation. Proteins that were significantly changed by OSM treatment are shown in *green* (*t* test, *p* < 0.05).

Having established that lysosomes from OSM-treated EpH4 cells could be isolated using our purification method, we then investigated the effect of Stat3 activation on the lysosomal proteome. Lysosome membrane preparations from three independent biological replicates of vehicle and OSM-treated cells were submitted for LC-MS/MS analysis. For each replicate a corresponding unlabeled (no iron particles) control was also included to filter out nonspecific contaminants. Prior to MS analysis, lysosomes purified from OSM and control (vehicle)-treated cells were assessed for lysosomal membrane enrichment (Fig. 4*A*) and purity (Fig. 4*B*). Importantly, Western blot analysis of iron-labeled LM preparations revealed nearly undetectable levels of contamination by other organelles (Fig. 4*B*). However, we observed low levels of COX IV, a mitochondrial marker, in one of the three replicates (Fig. 4*B*), which was derived either from contaminating mitochondria or from lysosomal mitophagic cargo. A label-free, exponentially modified protein abundance index (emPAI)-based semiquantitative proteomics approach was used to profile OSM-induced changes in protein abundance (see “Experimental procedures”). Raw data for all samples are shown in Table S4. MS analysis of three independent biological repeats (three OSM and corresponding vehicle control samples) resulted in the identification of 35,076 spectra within a 1% false discovery rate (0.66% decoy FDR) and an average spectrum identification rate of over 24% (Table S5). Coalescence of redundant identifications from repeat and overlapping peptides resulted in a list of 644 unique proteins at a probability of over 99%, which contained at least three identified peptides (established at greater than 95% probability), within a false discovery rate of less than 5% (Fig. 4*C* and Tables S4 and S6). Proteins that were also identified in the corresponding unlabeled (no magnetic particles) control sample for each independent replicate, not belonging to the endocytic-lysosomal pathway, were removed from this list. In addition, known common contaminants (35) were deleted as described above. Exceptions to this rule were considered if their representations changed significantly with OSM treatment, *e.g.* keratin 8, which is a definitive marker of luminal mammary epithelial cells. This resulted in a subset of 447 proteins (Fig. 4*C* and Table S7); 320 of these 447 proteins were identified in at least five of the six preparations (3× vehicle-treated and 3× OSM-treated samples) (Table S8). Pathway analysis (KEGG (37)) on this more stringent subset validated the lysosomal nature of the proteins identified, which included numerous known lysosomal components (Fig. 4*D* and Table S9*A*). A significant enrichment was also observed for components involved in phagocytosis. This was not unexpected given that the ultimate destination of phagosomes is the lysosomal compartment. Moreover, GO term distribution analysis for the cellular component confirmed the lysosomal and vesicular membrane localization of the identified proteins (Fig. 4*E* and Table S9*B*). In vehicle- and OSM-treated samples, 3 and 21 proteins, respectively, were identified exclusively (Fig. 4*C* and Table 2).

**Table 2:** **Proteins identified by MS analysis in control (vehicle) or OSM-treated lysosomal preparations only** These data are associated with Fig. 4 and Tables S4, S6, and S7.

Accession number (UniProt)	Gene symbol	Protein name
**Vehicle only**		
Q8VDV8	*Mitd1*	MIT domain–containing protein 1
Q9WVE8	*Pacsin2*	Protein kinase C and casein kinase substrate in neurons protein 2
Q8BHL4	*Gprc5a*	Retinoic acid–induced protein 3

**OSM only**		
Q80X90	*Flnb*	Filamin-B
Q9EPR5	*Sorcs2*	VPS10 domain–containing receptor SorCS2
Q8BKG3	*Ptk7*	Inactive tyrosine-protein kinase 7
P40124	*Cap1*	Adenylyl cyclase–associated protein 1
Q91V01	*Lpcat3*	Lysophospholipid acyltransferase 5
P48678	*Lmna*	Prelamin-A/C
Q61738	*Itga7*	Integrin α-7
Q91V92	*Acly*	ATP-citrate synthase
Q91VS7	*Mgst1*	Microsomal glutathione *S*-transferase 1
Q8BU30	*Iars*	Isoleucine–tRNA ligase, cytoplasmic
O70309	*Itgb5*	Integrin β-5
Q80VQ0	*Aldh3b1*	Aldehyde dehydrogenase family 3 member B1
P35821	*Ptpn1*	Tyrosine-protein phosphatase non-receptor type 1
O70133	*Dhx9*	ATP-dependent RNA helicase A
Q9DBG3	*Ap2b1*	AP-2 complex subunit β
P14901	*Hmox1*	Heme oxygenase 1
Q6P9J9	*Ano6*	Anoctamin-6
Q8BTM8	*Flna*	Filamin-A
Q61739	*Itga6*	Integrin α-6
P97384	*Anxa11*	Annexin A11
Q62261	*Sptbn1*	Spectrin β-chain, non-erythrocytic 1

Although the lysosomal nature of most proteins (408 of 447) remained similar between vehicle- and OSM-treated preparations, a subset of 39 proteins (∼ 9%) showed more significant changes in abundance between the different conditions (Fig. 4*C*, Table 3, and Table S10). A striking decrease in lysosomal enzymes, including galactocerebrosidase, β-hexosaminidase, and lysosomal α-mannosidase, was observed with OSM treatment (Fig. 4*F*, Table 3, and Table S10), suggesting either that Stat3 affects the distribution of these enzymes or, more likely, that these have been depleted from the lysosomes as a result of LMP. OSM stimulation also resulted in the increased representation of cell surface proteins and cytoskeletal components such as integrins, annexins, and flotillins (Fig. 4*F*, Table 3, and Table S10). Interestingly, this suggests that endocytosis, and the subsequent transport and localization of cargo into the lysosomal compartment, is stimulated upon Stat3 activation, supporting our previous observations *in vivo* (20).

**Table 3:** **Proteins represented differentially in OSM-treated lysosomal preparations** Sixteen (italic) and 23 (bold) proteins were differentially represented in control and OSM-treated samples (depicted in Fig. 4*C*). Many lysosomal enzymes were decreased by OSM, whereas the lysosomal localization of cell surface and cytoskeletal components were increased. Proteins that were absent/nearly absent from the comparator sample (Infinite (INF) or 0) are not plotted in Fig. 4*F*.

Protein name	Gene symbol	Accession no.	*p* value*^a^*	-Fold change*^b^*
MIT domain–containing protein 1 OS	*Mitd1*	Q8VDV8	0.034	*0*
Epoxide hydrolase 1 OS	*Ephx1*	Q9D379	0.015	*0.06*
Galactocerebrosidase OS	*Galc*	P54818	0.0021	*0.09*
Palmitoyl-protein thioesterase 1 OS	*Ppt1*	O88531	0.00089	*0.1*
Acid ceramidase OS	*Asah1*	Q9WV54	0.014	*0.1*
E3 ubiquitin-protein ligase RNF13 OS	*Rnf13*	O54965	0.043	*0.1*
CD82 antigen OS	*Cd82*	P40237	0.0091	*0.2*
β-Hexosaminidase subunit beta OS	*Hexb*	P20060	0.022	*0.2*
Lysosomal α-mannosidase OS	*Man2b1*	O09159	0.014	*0.3*
IST1 homolog OS	*Ist1*	Q9CX00	0.019	*0.3*
β-Glucuronidase OS	*Gusb*	P12265	0.047	*0.3*
Lactadherin OS	*Mfge8*	P21956	0.0067	*0.4*
Ras-related protein Rab-18 OS	*Rab18*	P35293	0.025	*0.4*
Claudin-3 OS	*Cldn3*	Q9Z0G9	0.011	*0.5*
Retinoid-inducible serine carboxypeptidase OS	*Scpep1*	Q920A5	0.014	*0.5*
Sideroflexin-1 OS	*Sfxn1*	Q99JR1	0.046	*0.5*
Lysosome-associated membrane glycoprotein 2 OS	*Lamp2*	P17047	0.003	**1.5**
Integrin β-1 OS	*Itgb1*	P09055	0.017	**2.2**
Monocarboxylate transporter 1 OS	*Slc16a1*	P53986	0.022	**2.5**
Basigin OS	*Bsg*	P18572	0.0017	**3.1**
Importin subunit β-1 OS	*Kpnb1*	P70168	0.0026	**3.5**
Annexin A1 OS	*Anxa1*	P10107	0.0051	**4.3**
ADP-ribosyl cyclase 2 OS	*Bst1*	Q64277	0.022	**4.5**
Catenin δ-1 OS	*Ctnnd1*	P30999	0.013	**4.9**
Annexin A6 OS	*Anxa6*	P14824	0.0025	**5.8**
Junction plakoglobin OS	*Jup*	Q02257	0.023	**8.1**
Talin-1 OS	*Tln1*	P26039	0.032	**8.5**
Flotillin-1 OS	*Flot1*	O08917	0.036	**8.7**
Keratin, type II cytoskeletal 8 OS	*Krt8*	P11679	0.0016	**9.3**
T-complex protein 1 subunit β OS	*Cct2*	P80314	0.018	**9.6**
Vinculin OS	*Vcl*	Q64727	0.027	**12**
Protein-glutamine γ-glutamyltransferase 2 OS	*Tgm2*	P21981	0.044	**14**
Microsomal glutathione *S*-transferase 1 OS	*Mgst1*	Q91VS7	0.0049	**INF**
Filamin-A OS	*Flna*	Q8BTM8	0.0056	**INF**
Integrin α-6 OS	*Itga6*	Q61739	0.027	**INF**
Annexin A11 OS	*Anxa11*	P97384	0.028	**INF**
Filamin-B OS	*Flnb*	Q80X90	0.041	**INF**
Integrin α-7 OS	*Itga7*	Q61738	0.041	**INF**
Inactive tyrosine-protein kinase 7 OS	*Ptk7*	Q8BKG3	0.042	**INF**

*^a^* Determined by *t* test.

*^b^* OSM/vehicle as measured by emPAI.

**Figure 5.**
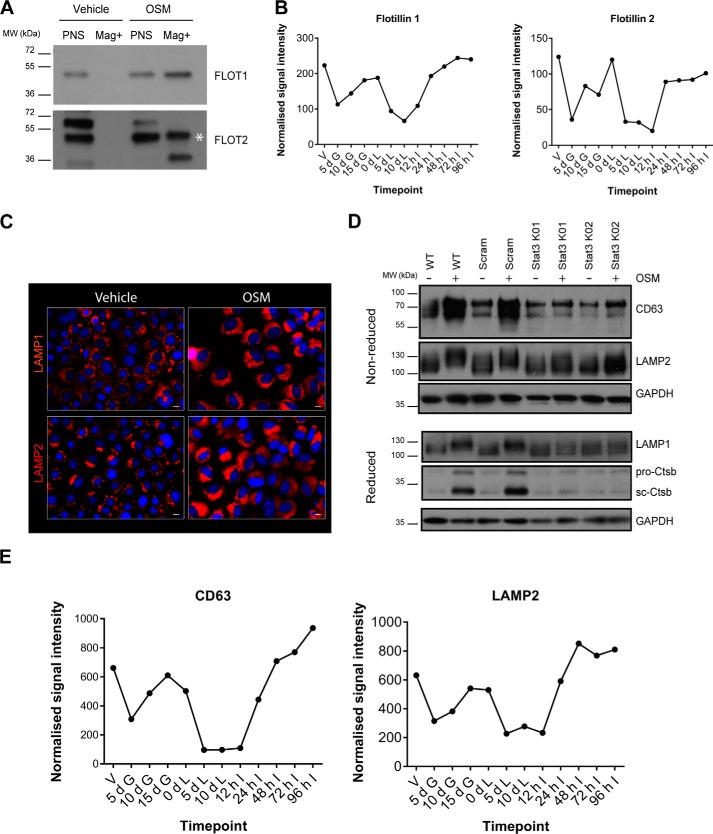
**Stat3-mediated regulation of the lysosomal compartment.** *A*, validation by Western blot analysis of the lysosomal localization of flotillin 1 and flotillin 2 using iron nanoparticles to isolate EpH4 lysosomes (Mag+) after 72 h of OSM stimulation. *White asterisk*, indicates the expected molecular weight of flotillin 2. Representative blots of *n* = 3 (flotillin 1) and *n* = 2 (flotillin 2) independent experiments are shown. *B*, microarray analysis of 12 different timepoints during the mammary gland pregnancy cycle showing the involution-related expression profiles of flotillin 1 and flotillin 2. *V*, virgin; *d G*, days gestation; *d L*, days lactation; *h I*, hours involution. *C*, fluorescence microscopy of LAMP1 and LAMP2 immunostaining in EpH4 cells treated with OSM for 72 h. *Scale bars*: 10 μm. *D*, Western blot analysis of OSM-induced changes to the lysosomal proteins LAMP1, LAMP2, CD63, and cathepsin B in whole-cell lysates from normal (WT) and two independent Stat3 KO EpH4 cell lines after 72 h of stimulation. Proteins were separated by SDS-PAGE under reducing or non-reducing conditions as indicated. *pro-Ctsb*, pro form of cathepsin B; *sc Ctsb*, single-chain cathepsin B; *Scram*, scrambled sgRNA control EpH4 cells. *E*, microarray analysis of 12 different timepoints during the mammary gland pregnancy cycle showing the involution-related expression profiles of CD63 and LAMP2.

To validate these results, immunoblot analysis of lysosomal protein preparations, used for the MS analysis, was undertaken. Flotillins are interesting candidates, as they are involved in endocytosis and membrane trafficking and form clusters in plasma membrane lipid rafts (42, 43). Flotillin 1 was significantly enriched in lysosomes isolated from OSM-stimulated cells (Fig. 4*F* and Table 3), and although not statistically significant, the related protein, flotillin 2, also showed an increased representation in OSM-treated lysosomes (Fig. 4*F*). Although flotillin 1 and flotillin 2 were undetectable in lysosomal preparations from untreated EpH4 cells, OSM stimulation of Stat3 activity resulted in the appearance of considerable amounts of both of these proteins in the lysosomal compartment (Fig. 5*A*). This was not surprising, as flotillin 2 has been observed previously to traffic from the plasma membrane to endosomes (42, 43), which may subsequently fuse with lysosomal structures. Further support for the endocytosis of flotillins in response to Stat3 is provided by the observation that OSM treatment of EpH4 cells for only 24 h resulted in increased internalization of flotillin 2 puncta in EpH4 cells (Fig. S2).

Annexins A1, A6, and A11 were also enriched in lysosomal fractions following OSM stimulation (Table 3). Our previous microarray data of a mammary gland developmental cycle (44) revealed the down-regulation of these annexins during lactation (Fig. S3). Interestingly, although expression levels of annexin A6 remained fairly consistent across all lactation and involution timepoints, a substantial increase in the expression of annexins A11 and A1 was observed during involution (Fig. S3). This pattern is similar to that of flotillins 1 and 2 (Fig. 5*B*), suggesting that all of these membrane proteins may play significant roles in the regression of the mammary gland. Indeed, mRNA expression of both *Flot1* and *Flot2* is down-regulated ∼2-fold in the mammary glands of Stat3 knockout mice at 24 h involution (Table S11). In addition, the glutathione *S*-transferase Mgst1 was also identified in lysosomal preparations purified from OSM-treated EpH4 cells (Tables 2 and 3). Mgst1 is a detoxifying enzyme and therefore may be up-regulated in response to iron nanoparticle uptake. However, as it was present only in the OSM-treated samples, it is tempting to speculate that it could be induced to protect against the effects of LMP. Indeed, an increase in Mgst1 and the related protein Mgst3 was observed during mammary gland involution (Fig. S3), suggesting that they may possess important functions during lysosome-mediated cell death *in vivo*.

Although a significant number of known lysosomal membrane proteins were identified by MS analysis, the relative yield of some peptides (including from abundant proteins such as LAMP1 and LAMP2) was lower than expected, similar to what was observed in previous studies (34). One possible explanation is that the heavily glycosylated nature of many lysosomal membrane proteins may interfere with MS analysis. To address this possibility, lysosomal membrane samples were deglycosylated using peptide-*N*-glycosidase F (PNGase F) prior to proteomic analysis. PNGase F is an amidase that cleaves between the innermost *N*-acetylglucosamin**e (**GlcNAc) and asparagine residues of oligosaccharides from *N*-linked glycoproteins (45). PNGase F treatment resulted in the efficient removal of *N*-linked glycans from LAMP2 proteins, as shown by the appearance of a sharp band at ∼30 kDa by immunoblot analysis (Fig. S4). To determine whether the representation of heavily glycosylated proteins is improved after deglycosylation, PNGase F enzyme–treated and untreated lysosomal membrane samples were analyzed by LC-MS/MS. Western blot analysis of various organelle markers was performed to check the purity of the preparations as described previously (Fig. S4). 955 and 718 proteins were identified in control and enzyme-treated lysosomal membrane samples, respectively (Table S12). Comparative analysis of heavily glycosylated proteins, such as LAMP1, LAMP2, LIMP2, and CD63/LIMP1, revealed that deglycosylation increased the number of unique peptide sequences identified for these proteins, vastly improving their representation and identification by LC-MS/MS (Table S13). Of note, deglycosylation did not appear to have a detrimental impact on the identification of lysosomal membrane proteins, with the majority higher ranked in PNGase F-treated samples (Table S13). Therefore, future proteomic experiments investigating lysosomal membrane composition will likely benefit from utilizing deglycosylating approaches prior to LC-MS/MS.

### Stat3-mediated regulation of the lysosomal compartment

To investigate the biological relevance of OSM-induced changes to the lysosomal compartment, we used CRISPR/Cas9 technology to ablate Stat3 in EpH4 cells. Mass-spectrometry analysis revealed that the lysosomal membrane protein LAMP2 is up-regulated in response to OSM stimulation of Stat3 activity (Fig. 4*F* and Table 3). Indeed, LAMP2 and LAMP1 immunostaining in EpH4 cells clearly showed enhanced lysosomal biogenesis in response to OSM, with lysosomes becoming increasingly perinuclear in distribution (Fig. 5*C*). Surprisingly, we found that the deletion of Stat3 abrogated the increase in the apparent molecular weight of LAMP1 and LAMP2 that occurs in response to OSM treatment (Figs. 3*E* and 5*D*), suggesting a role for this post-translation modification of LAMP1 and LAMP2 in lysosome function. Importantly, a similar shift in mobility is observed during involution of the mouse mammary gland (30) and is likely due to a change in glycosylation. Stat3 also up-regulates the expression of the lysosomal protease cathepsin B and the endolysosomal membrane protein CD63 (Fig. 5*D* and Table S11). At a transcriptional level, both *CD63* and *Lamp2* mRNAs are down-regulated during lactation and rise sharply during involution (Fig. 5*E*). Together, these data suggest that Stat3 directly modulates lysosomal membrane constituents to induce lysosomal leakiness and consequent cell death.

## Discussion

The lysosome is enjoying a renaissance as a signaling center and monitor of cellular nutritional status (46). Although microarray studies are very useful in highlighting transcriptional changes in response to transcription factors such as Stat3, they are of little value in determining the functional downstream consequences of altered protein expression. Having determined a role for Stat3 in lysosomal function, we aimed to characterize this at the level of the lysosomal membrane proteome to shed further light on the mechanism of LMP in mammary epithelial cells.

In the past decade, an increased knowledge of the lysosomal proteome has been acquired through the application of MS (8, 14, 22, 34). However, although numerous lysosomal membrane proteins have thus far been identified using density centrifugation (14, 34), these data do not fully correlate with the plethora of known and crucial functions of the lysosomal membrane and its constituent proteins and enzymes. This failure to detect all lysosomal membrane proteins, coupled with the contamination inherent in lysosome enrichment approaches used to date, prompted us to develop an alternative method to enrich lysosomal membrane proteins. To circumvent alterations in the size or buoyant density of lysosomes in response to changes in cellular status, we utilized a different approach that is independent of these features. Labeling and subsequent isolation of lysosomes by uptake of magnetic nanoparticles resulted in highly pure lysosomal membrane preparations being obtained. Although proteins that reside in other organelles were not detected by immunoblotting and MitoTracker^TM^ analysis, a number of mitochondrial and other organellar proteins were detected by MS. This could be due to contamination, but we suggest that it is more likely the consequence of autophagy, which we have shown previously to be induced in EpH4 cells, particularly in response to OSM (38). Indeed, we observed a considerable number of degradative structures present even in control, non-OSM–stimulated cells (Fig. 3) (20). Functional analysis of our MS data corroborated the lysosomal nature of the samples and provided valuable insights into membrane trafficking events as well as lysosomal function. Importantly, although heavily glycosylated proteins such as LAMP1, LAMP2, CD63/LIMP1, and LIMP2 were detected, they were relatively under-represented. This is likely due to glycosylation-mediated changes in mass and therefore the time of flight, which has an impact on the resolution of the peptide spectrum acquired (47). We show here that prior deglycosylation treatment considerably improves the representation of these proteins (Table S13) and suggest that future proteomics experiments should consider utilizing this approach.

We analyzed the lysosomal proteome in both intact and leaky lysosomes, the latter treated with OSM to induce Stat3 activation and subsequent LMP. Interestingly, OSM treatment resulted in a clear loss of lysosomal enzymes, probably as a consequence of LMP and leakage of proteases to the cytosol. In contrast, OSM-induced elevated cargo levels, probably as a consequence of the increased endocytosis and down-regulation of cell surface receptors and adhesion complexes that are subsequently delivered to the lysosome. Observed changes in cytoskeletal proteins may be relevant to lysosomal localization, which in turn influences internal pH (48) with possible effects on protease functions and LMP. Intriguingly, in support of this notion, OSM stimulation resulted in increased perinuclear distribution of lysosomes in EpH4 cells (Figs. 3*D* and 5*C*).

Interesting candidates for further characterization include flotillins 1 and 2. Data from this study show that OSM treatment causes trafficking of these proteins to the degradative endolysosomal pathway. Flotillins (or reggies) form clusters in lipid rafts and have been shown to regulate trafficking of α5 and β1 integrins and thus the formation of focal adhesions (49). We speculate that OSM treatment would induce cells to detach, with consequent turnover of focal adhesions and trafficking of integrins to the lysosome. Indeed, mRNA expression of both *Flot1* and *Flot2* is down-regulated ∼2-fold in the mammary glands of Stat3 knockout mice at 24 h involution (Table S11). Their roles in regulating mammary cell death during involution *in vivo*, therefore, warrants further investigation.

Other interesting candidates for validation include annexins A1 (Anxa1), A6 (Anxa6), and A11 (Anxa11). Annexins are a family of highly conserved proteins that bind to negatively charged phospholipids and membranes in a calcium-dependent manner (50). Studies in annexin knockout mice suggest that annexin A1 fulfils multiple roles, including trafficking at the plasma membrane and in the endocytic compartment (50). Annexin A6 is also involved in membrane organization and the distribution of proteins in rafts (51). Recently, annexin A11 has been shown to physically associate with a component of the coat protein complex II (COPII) machinery that is associated with the ER and controls the transport of transmembrane cargoes from the ER to the Golgi (52). Thus, the changes we observed in the lysosomal levels of these three annexins could be a reflection of the elevated vesicle formation induced by OSM.

Finally, using CRISPR/Cas9 technology to ablate Stat3 in EpH4 cells, we reveal a direct role for Stat3 signaling in the regulation of lysosomal proteins such as cathepsin B, CD63, LAMP1, and LAMP2. Surprisingly, we show that Stat3 regulates the glycosylation status of LAMP1 and LAMP2 in response to OSM treatment in EpH4 cells (Fig. 5*D*), mimicking that observed during involution of the mouse mammary gland (30). An intriguing possibility is that Stat3 could induce the expression of fucosyltransferases in mammary epithelial cells, as LIF/Stat3 has been shown to regulate expression of α1,2-fucosyltransferases FUT1 and FUT2, which transfer fucoses onto the terminal galactose of *N*-acetyllactosamine (53). It has been shown recently that FUT1 regulates the subcellular localization of lysosomes, with FUT1 knockdown inducing perinuclear accumulation (54) similar to that observed upon OSM stimulation (Figs. 3*D* and 5*C*). Together, our data suggest that Stat3 activation induces the endocytosis of plasma membrane constituents (including flotillins and annexins) consequently resulting in a dramatic increase in endolysosomal trafficking and the subsequent enhancement of the lysosomal system.

In summary, this study has refined the utility of magnetic iron nanoparticles for the isolation of highly pure lysosomal membrane preparations for downstream proteomic analysis. Notably, this method can be applied to the purification of leaky lysosomes undergoing membrane permeabilization, revealing valuable insights into the cellular trafficking events that may contribute to Stat3-mediated cell death. Vacuolization is implicated in driving cell death in a variety of organisms, including plant (55) and *Dictyostelium discoideum* (56) cells. Intriguingly, an activated form of the H-Ras oncoprotein was recently reported to induce extensive vacuolization and subsequent cell death in cultured cancer cells (57). Thus, our method may be utilized more widely to investigate the lysosomal proteome in contexts associated with lysosomal perturbations, such as cancer, neurodegenerative diseases, and lysosomal storage disorders. An improved understanding of the mechanisms driving lysosomal membrane permeabilization is pivotal for developing new and effective therapies against the many disorders associated with aberrant lysosomal function.

## Experimental procedures

### Cell culture

EpH4 cells (58) were maintained in DMEM (Life Technologies) supplemented with 10% FCS (Sigma) at 37 °C in a humidified atmosphere of 5% CO_2_. For OSM stimulation, cells were stimulated at 50% confluency with a final concentration of 25 ng ml^−1^ recombinant mouse oncostatin M (495-MO, R&D Systems) or carrier (0.0001% BSA in PBS) in DMEM supplemented with 1% FCS. Medium was renewed with fresh OSM (in 1% FCS/DMEM) after 48 h. Cells were harvested 72 h after stimulation. Cytotoxicity was assessed by trypan blue exclusion.

### CRISPR/Cas9 Stat3 KO cell line generation

Two SpCas9 guide sequences targeting the mouse Stat3 gene were designed using the sgRNA scoring algorithm from Doench *et al*. (59). These were cloned into the lentiCRISPRv2 plasmid, lentivirus was produced, and EpH4 cells were transduced and selected using puromycin according to previously described protocols (60, 61). Using different guide sequences, two independent Stat3KO lines were derived and maintained in the pooled state (as single-cell cloning of EpH4 cells would likely lead to clonal artifacts). Stat3KO1 was created from virus expressing the guide sequence 5′-GTACAGCGACAGCTTCCCCA-3′ and Stat3KO2 from virus expressing the guide sequence 5′-GGAACTGCCGCAGCTCCATG-3′. As these two lines use different guide sequences, they act as off-target controls for one another, and so any phenotypes observed can be attributed to the loss of Stat3. Deletion of Stat3 was confirmed by TIDE analysis (62) and Western blotting. To further control for any effects of lentiviral transduction and SpCas9 expression, virus expressing a “scrambled” guide (5′-AAATTAAATTTAATTTAAAG-3′) that does not target any mouse coding sequences was also used.

### Ferrofluid labeling and purification of lysosomes

Lysosomes from EpH4 cells were purified using magnetic iron nanoparticles (EMG-508, Ferrotec) as described previously (20). Briefly, EpH4 cells were seeded at a density of 3 × 10^6^ or 1 × 10^6^ for OSM stimulation in 15-cm tissue culture plates (catalog no. 168381, Nunc). The following day, or after 48 h of OSM stimulation, cells were labeled for 4 h in iron nanoparticle–containing media (EMG series 508, FerroTec, 1:100) followed by a 20-h chase period in clean medium (including fresh OSM in the case of stimulation experiments). Cells were then scraped in PBS and pelleted in a tabletop centrifuge (300 rpm at 4 °C for 3 min), and the pellet was homogenized in a tight-fitting hand-held homogenizer (5 strokes) in 700 μl of subcellular fractionation buffer (20 mm HEPES-KOH, 250 mm sucrose, 10 mm KCl, 1.5 mm MgCl_2_, 1 mm EDTA, 1 mm EGTA, 8 mm dithiothreitol, and Complete protease inhibitor (Roche) at pH 7.5). The homogenate was spun at 750 × *g* (3500 rpm at 4 °C for 10 min) to remove nuclei and other debris followed by a second 750 × *g* spin to ensure complete removal of contaminating heavy membranes. The resulting supernatant (post-nuclear supernatant (PNS)) was transferred into a clean tube, loaded onto a magnetic rack, and incubated for 1 h at 4 °C on a rocker. A 50-μl sample of PNS was retained for analysis. Following incubation, tubes were left on the magnet, and the supernatant (SN) was removed (retaining 50 μl for analysis). Tubes were washed three times with 1 ml of subcellular fraction buffer. Following the addition of the last wash, tubes were removed from the magnet and spun at 12,000 rpm (13,800 × *g* at 4 °C for 15 min) to pellet magnetite-containing lysosomes. To limit contamination by proteins nonspecifically sticking to the Eppendorf microcentrifuge tube, the pellets were resuspended, combined in a final volume of 200 μl of fractionation buffer, and transferred into a clean Eppendorf microcentrifuge tube. The samples were then pelleted at 13,800 × *g* for a further 15 min at 4 °C along with the PNS and SN samples.

### Fractionation of purified lysosomes

To obtain “total” lysosomal lysates, whole lysosomes purified as described above were incubated with 0.1% Triton X-100 (VWR International) for 10 min on ice, vortexed intermittently four times, and spun at 12,000 rpm (13,800 × *g* at 4 °C for 15 min). The supernatant was transferred into new tubes. To separate the lysosomal content from the lysosomal membrane fraction, the isolated lysosomes were resuspended in 5 mm Tris buffer, pH 7.5, incubated on ice for 30 min or frozen in liquid nitrogen, and then thawed at 37 °C five times. Lysosomal membranes were pelleted by spinning at 15,000 × *g* for 30 min at 4 °C. The supernatant (lysosomal content (LC)) was transferred into a new tube. The pellet was resuspended in 50 μl of 0.1% Triton X-100 in PBS, incubated for 10 min on ice, and intermittently vortexed four times. After spinning at 17,000 × *g* for 10 min at 4 °C, the supernatant (LM) was transferred into fresh tubes.

### Immunoblotting

PNS and SN samples were pelleted, resuspended in 50 μl of modified radioimmune precipitation assay buffer (50 mm Tris, 1% Nonidet P-40, 0.25% sodium deoxycholate, 150 mm NaCl, 1 mm EGTA, and Complete protease inhibitor), and lysed on ice for 30 min with intermittent vortexing. Samples were then spun at 12,000 rpm (13,800 × *g* at 4 °C for 15 min), and the supernatant was retained for Western blot analysis. Protein samples (made up in modified radioimmune precipitation assay buffer with 0.1% Triton X-100 or 5 mm Tris as described above) were denatured and resolved on SDS-polyacrylamide gels. Proteins were separated by SDS-PAGE under reducing conditions unless stated otherwise. Immunoblotting was performed using standard techniques, and antibody detection was achieved with enhanced chemiluminescence reagent (ECL, GE Healthcare). The following primary antibodies were used: rat anti-LAMP2 (ab13524, Abcam, 1:1,000), goat anti-cathepsin L (AF1515, R&D Systems, 1:1,000), goat anti-cathepsin B (AF965, R&D Systems, 1:1,000), mouse anti-COX IV (ab33985, Abcam, 1:1,000), rabbit anti-phosphatidylinositol 3-kinase, p85 (06-496, Millipore, 1:1000), rabbit anti-Stat3 (sc-482, Santa Cruz Biotechnology, 1:1000), rabbit anti-phospho-Stat3 (9131, Cell Signaling, 1:1000), mouse anti-ERp57 (sc-23886, Santa Cruz Biotechnology, 1:400), rabbit anti-GAPDH (5174, Cell Signaling, 1:1000), goat anti-histone H3 (sc-8654, Santa Cruz Biotechnology, 1:1000), rabbit anti-Rab5 (2143, Cell Signaling, 1:1000), rat anti-LAMP1 (ID4B, Developmental Studies Hybridoma Bank, 1:200), rat anti-CD63 (NVG-2, 143901, Biolegend, 1:1000), rabbit anti-flotillin 1 (18634, Cell Signaling Technology, 1:800), and rabbit anti-flotillin 2 (HPA0013961, Atlas Antibodies, 1:200). All horseradish peroxidase-conjugated secondary antibodies were from Dako.

### Cathepsin activity assay

10 μl of lysate was added to a total of 200 μl of cathepsin reaction buffer (50 mm sodium acetate, 8 mm EDTA, 8 mm dithiothreitol, and 1 mm Pefabloc subcellular fractionation buffer at pH 6). Cathepsin B and L activity was measured with the fluorescent substrate Z-Phe-Arg-AMC (50 μm, Merk Millipore) in a Synergy HT multi-detection microplate reader (excitation 380 nm and emission 442 nm, BioTek). Fluorescence was measured immediately every minute for 1 h at 37 °C. The initial rates of cathepsin B and L activity were determined from the linear part of the resulting curve and normalized to total cathepsin activity obtained from Triton X-100–extracted lysates. Cathepsin activity reaction buffer alone or containing 50 μm Z-Phe-Arg-AMC served as the control. The assay was performed in duplicate or triplicate per condition in a 96-well flat-bottom microtiter plate (Thermo Scientific).

### β-Glucuronidase assay

10 μl of lysate was added to 125 μl of sodium acetate/acetic acid buffer (0.1 m sodium acetate and 0.1 m acetic acid, pH 4.8) containing 10 mm of the fluorescent substrate 4-methylumbelliferone-β-d-glucuronide (4-MU-Glu, Biosynth) on ice. Samples were incubated for 30 min at 37 °C, and the reaction was stopped subsequently by the addition of 100 μl of glycine/carbonate buffer (0.17 m glycine and 0.17 m sodium carbonate, pH 9.8) on ice. Fluorescence was measured in a Synergy HT multi-detection microplate reader (excitation 380 nm and emission 442 nm, BioTek). Sodium acetate/acetic acid buffer alone or containing 10 mm 4-MU-Glu served as the control. The assay was performed in duplicate or triplicate per condition in a 96-well flat-bottom microtiter plate (Thermo Scientific).

### FITC-dextran and MitoTracker staining of lysosomal preparations

EpH4 cells were seeded at a density of 3 × 10^6^ in 15-cm Nunc cell culture dishes (Thermo Scientific). Plates were labeled for 2 h with 2.5 mg/ml 10-kDa FITC-dextran (Sigma) in 10% DMEM containing 10% ferrofluid in BSA/PBS (+) or in FITC-dextran–containing medium only (minus iron nanoparticles (−)). Cells were washed three times in PBS followed by a 2-h chase period in 10% DMEM prior to labeling of the mitochondria with 500 nm MitoTracker (MitoTracker Red FM, Invitrogen) in 10% DMEM for 30 min. Plates were then washed three times in PBS, and the cells were scraped into 500 μl of PBS and spun at 900 × *g* for 3 min at 4 °C. Cell pellets were resuspended in 700 μl of fractionation buffer containing Complete protease inhibitors and homogenized with five strokes in a Dounce homogenizer. To obtain the PNS, the homogenate was spun at 800 × *g* for 10 min at 4 °C. The PNS was transferred into a new tube, and 50 μl was retained for analysis (PNS^−/+^). The PNS was incubated on a magnetic rack for 1 h at 4 °C on a rocker. Post-incubation SN was removed, and 50 μl was kept for analysis (SN^−/+^). The tubes were washed three times with 1 ml of cold fractionation buffer on the magnetic rack. Following the last wash, the tubes were removed and spun at 13,800 × *g* for 15 min at 4 °C. The lysosomal pellet (LP^−/+^) was resuspended in 25 μl of fractionation buffer. 10 μl of the magnetic nanoparticle-labeled and unlabeled PNS, SN, and lysosomal pellets was mixed with 10 μl of PBS/glycerol (v/v 50:50), dropped onto a microscope slide, and covered by a coverslip for imaging using a Zeiss Axioplan 2 microscope and a Plan-Apochromat 63x/1.4 oil objective (Zeiss).

### Immunofluorescence and LysoTracker Red staining

EpH4 cells were seeded on glass coverslips or ibidi 35-mm glass-bottom dishes and stimulated with OSM as described above. After OSM stimulation cells were fixed with 4% paraformaldehyde for 5 min at 37 °C and subsequently permeabilized with 0.5% v/v TritonX-100 in PBS for 10 min at room temperature. Cells were blocked in 10% normal goat serum (Sigma) in PBS for 1 h at room temperature and then incubated with primary antibody in PBS for 1 h at room temperature or overnight at 4 °C. Primary antibodies used were rat anti-LAMP2 (GL2A7, ab13524, Abcam, 1:200), rat anti-LAMP1 (ID4B, Developmental Studies Hybridoma Bank, 1:200), and rabbit anti-flotillin 2 (HPA0013961, Atlas Antibodies, 1:200). Cells were then washed in PBS followed by secondary antibody staining (Cy3 goat anti-rat IgG, Life Technologies, 1:500; Alexa Fluor-488 goat anti-rabbit IgG, Invitrogen, A11008, 1:500; or Alexa Fluor-647 goat anti-rat IgG, Life Technologies, A21247, 1:500) for 1 h at room temperature. Nuclei were counterstained with Hoechst 33342 (Sigma Aldrich, 1:1000). Cells were stained with LysoTracker Red DND-99 (Life Technologies) at 1:10,000 for 30 min at room temperature according to the manufacturer's instructions. Images were acquired using a Zeiss Axioplan 2 microscope (Zeiss, Jena, Germany) or a Leica TCS SP8 inverted confocal microscope. Image reconstructions were generated using ImageJ (version 1.50c, National Institutes of Health) (63).

### Transmission election microscopy

EpH4 cell lysosomes were isolated using the magnetic nanoparticle protocol and adsorbed to a TEM grid, followed by two wash steps in H_2_O to remove any remaining buffer salts. Samples were either imaged directly using a Tecnai G2 electron microscope or negatively stained with 1% uranyl acetate beforehand.

### Silver staining of protein gels

Protein samples were run on 4–15% Mini-Protean TGX gels (Bio-Rad) and fixed in 45% methanol, 1% v/v, in ddH_2_O for 30 min. After washing in ddH_2_O, the gels were sensitized with 0.02% sodium thiosulfate (Na_2_S_2_O_3_) for 1 min and rinsed in ddH_2_O three times for 20 s. Next the gels were stained in ddH_2_O containing 0.2% silver nitrate (AgNO_3_) and 0.02% formaldehyde for 20 min. This was followed by rinsing the gels twice in ddH_2_O for 20 s and developing them for 3 min in 3% sodium carbonate (Na_2_CO_3_), 0.0005% sodium thiosulfate, 0.05% formaldehyde in ddH_2_O. The developed gels were rinsed twice in ddH_2_O for 20 s and blocked in 5% acetic acid.

### Mass-spectrometry methods

All MS experiments were undertaken at the Cambridge Centre for Proteomics (University of Cambridge). For all experiments submitted for MS analysis, a corresponding unlabeled (no magnetic particles) control sample was also analyzed. For the OSM-related experiments, three independent biological repeats (three OSM and corresponding vehicle control samples) were performed, and a corresponding unlabeled (no magnetic particles) control sample also was submitted for each independent replicate.

### Silver destaining and tryptic digest

After 1D gel electrophoresis, gel pieces were cut into 1–2–mm cubes and placed into 0.5-ml tubes. The gel pieces were incubated with 100 μl of silver-destaining solution (a 1:1 ratio of 27 mm potassium ferricyanide (K_3_[Fe(CN)_6_]) and 100 mm sodium thiosulphate (Na_2_S_2_O_3_) in high-performance liquid chromatography (HPLC) grade water) for 10 min at 37 °C. This step was repeated with fresh destaining solution until complete clarification of the gel pieces was obtained. Subsequently, the pieces were washed in HPLC-grade water prior to incubation in 100% acetonitrile at 37 °C until they appeared white and shrunken. The acetonitrile was removed, and any residual solution was evaporated by incubation at 37 °C for 10 min. To reduce the proteins, the gel pieces were incubated in 50 μl of 10 mm DTT made up in 100 mm ammonium bicarbonate (NH_4_HCO_3_) at 56 °C for 1 h. For alkylation 50 μl of 55 mm iodoacetamide (C_2_H_4_INO) made up in 100 mm ammonium bicarbonate was then added, and the gel pieces were incubated at room temperature in the dark for 45 min. The liquid was removed, 100 μl of 100 mm ammonium bicarbonate was added, and the gel pieces were incubated at 37 °C for 10 min. After removing the liquid, the gel pieces were incubated in 50% acetonitrile (C_2_H3N) in 100 mm ammonium bicarbonate at 37 °C for 10 min, which was then replaced by 100 μl 100% acetonitrile to dry the gel pieces. To evaporate the acetonitrile, the gel pieces were incubated further at 37 °C, ensuring that they were completely dry prior to tryptic digest. 50–60 μl of trypsin solution (10 ng/μl in 50 mm ammonium bicarbonate) was added to the gel pieces, and they were incubated overnight.

### LC-MS/MS analysis

After tryptic digestion, the supernatant was pipetted into a sample vial and loaded onto an autosampler for automated LC-MS/MS analysis. The pilot LC-MS/MS experiment was performed using a Dionex Ultimate 3000 RSLC nanoUPLC (Thermo Fisher Scientific Inc., Waltham, MA) system and a Q Exactive Orbitrap mass spectrometer (Thermo Fisher Scientific). All OSM-related and deglycosylation experiments were performed using a Waters NanoAcquity UPLC (Thermo Fisher Scientific) system and an Orbitrap Velos mass spectrometer (Thermo Fisher Scientific). For the pilot experiment using the Q Exactive mass spectrometer, separation of the peptides was performed by reverse-phase chromatography at a flow rate of 300 nl/min and a reverse-phase nano EASY-Spray column (Thermo Scientific PepMap C18, 2-μm particle size, 100-Å pore size, 75-μm i.d. × 50-cm length). Peptides were loaded onto a pre-column (Thermo Scientific PepMap 100 C18, 5-μm particle size, 100-Å pore size, 300-μm i.d. × 5-mm length) from the Ultimate 3000 autosampler with 0.1% formic acid for 3 min at a flow rate of 10 μl/min. After this time period, the column valve was switched to allow elution of peptides from the pre-column onto the analytical column. Solvent A consisted of water + 0.1% formic acid and solvent B consisted of 80% acetonitrile and 20% water + 0.1% formic acid. The linear gradient employed was 2–40% B in 30 min. The LC eluant was sprayed into the mass spectrometer by means of an EASY-Spray source (Thermo Fisher Scientific). All *m*/*z* values of eluting ions were measured in an Orbitrap mass analyzer set at a resolution of 70,000. Data-dependent scans (top 20) were employed to automatically isolate and generate fragment ions by higher-energy collisional dissociation (HCD) in the quadrupole mass analyzer, and measurement of the resulting fragment ions was performed in the Orbitrap analyzer set at a resolution of 17,500. Peptide ions with charge states of 2+ and above were selected for fragmentation.

For the nanoAcquity/Orbitrap Velos experiments, separation of the peptides was performed by reverse-phase chromatography using a Waters reverse-phase nano column (BEH C18, 75-μm i.d. × 250 mm, 1.7-μm particle size) at a flow rate of 300 nl/min. The peptides were initially loaded onto a pre-column (Waters UPLC Trap Symmetry C18, 180-μm i.d. × 20 mm, 5-μm particle size) from the nanoAcquity sample manager with 0.1% formic acid for 3 min at a flow rate of 10 μl/min. After this period, the column valve was switched to allow the elution of peptides from the pre-column onto the analytical column. Solvent A was water + 0.1% formic acid and solvent B was acetonitrile + 0.1% formic acid. The linear gradient employed was 3–40% B in 40 min (60 min total run time including wash and equilibration steps).

The LC eluant was sprayed into the mass spectrometer by means of a standard Thermo Scientific nanospray source. All *m*/*z* values of eluting ions were measured in the Orbitrap Velos mass analyzer set at a resolution of 30,000. Data-dependent scans (top 20) were employed to automatically isolate and generate fragment ions by collision-induced dissociation in the linear ion trap, resulting in the generation of MS/MS spectra. Ions with charge states of 2+ and above were selected for fragmentation. Post-run, all data were processed using Protein Discoverer (version 2.1, ThermoFisher).

All LC-MS/MS data generated from the pilot experiment, and deglycosylation experiments were converted to mgf files. These files were then submitted to the Mascot search algorithm (Matrix Science, London, UK) and searched against the UniProt mouse database (UniProt_Mouse_Oct13 10090_Oct2013 82208 sequences; 36,313,543 residues) using a fixed modification of carbamidomethyl (C) and variable modifications of oxidation (M) and deamidation (NQ). Peptide identifications were accepted if they could be established at greater than 95.0% probability.

For analysis of OSM-related experiments, all MS/MS data were converted to mgf files and submitted to the Mascot search algorithm (Matrix Science) and searched against the swissprot_2013_11 database (with a mouse taxonomy filter; 16,693 sequences). Mascot was searched with a fragment ion mass tolerance of 0.80 Da and a parent ion tolerance of 25 ppm. Trypsin was specified as the enzyme, and carbamidomethyl of cysteine was specified in Mascot as a fixed modification. Deamidation of asparagine and glutamine and oxidation of methionine were specified in Mascot as variable modifications. For protein identification, Scaffold (version Scaffold_4.7.2, Proteome Software Inc., Portland, OR) was used to validate MS/MS–based peptide and protein identifications. Peptide identifications were accepted if they could be established at greater than 95.0% probability by the Scaffold Local FDR algorithm. Protein identifications were accepted if they could be established at greater than 99.0% probability to achieve an FDR less than 5.0% and contained at least three identified peptides. Protein probabilities were assigned by the Protein Prophet algorithm (64). Proteins that contained similar peptides and could not be differentiated based on MS/MS analysis alone were grouped to satisfy the principles of parsimony. To estimate OSM-induced changes in protein abundance, the emPAI values of OSM and vehicle-treated samples were compared (65). emPAI provides an approximate and relative quantitation of the proteins in a mixture based on protein coverage by the peptide matches in a database search result. This method takes into account the fact that the more observed peptides will be generated for larger proteins and for proteins that have many peptides in the preferred mass range for mass spectrometry (65). Although emPAI and other similar spectral counting methods provide only semiquantitative information, they are highly correlated to protein abundance (65). EmPAI values were calculated in Scaffold, version 4.7.2, in addition to all comparative analyses of OSM and vehicle-treated samples and associated statistics.

### Deglycosylation of lysosomal proteins

One μl of 10× glycoprotein denaturing buffer (New England Biolabs) was added to 9 μl of lysosomal membrane proteins. The glycoproteins were denatured by heating to 95 °C for 10 min. Subsequently, 2 μl of 10× G7 reaction buffer (New England Biolabs), 2 μl of 10% Nonidet P-40 (New England Biolabs), and 1 μl of PNGase F (New England Biolabs) were added, and the reaction volume was made up to 20 μl with H_2_O. The reaction mix was incubated at 37 °C for 30 min. The deglycosylated protein samples were used for MS analysis or made up in 4× sample buffer and analyzed by Western blotting.

## Author contributions

B. L., T. J. S., K. S. L., and C. J. W. conceptualization; B. L., C. C. K., M.J.D., R.F., and J.A.H. data curation; B. L., C. C. K., T. J. S., and M. E. D. formal analysis; B. L. and C. J. W. supervision; B. L. validation; B. L., C. C. K., T. J. S., M. E. D., M. J. D., R. F., J. A. H., and C. J. W. investigation; B. L., C. C. K., T. J. S., M. E. D., M. J. D., R. F., and J. A. H. methodology; B. L. and C. J. W. writing-original draft; B. L. and C. J. W. project administration; B. L., T. J. S., M. J. D., K. S. L., and C. J. W. writing-review and editing; C. J. W. resources; C. J. W. funding acquisition.

## Supplementary Material

Supporting Information

## Footnotes

5 Please note that the JBC is not responsible for the long-term archiving and maintenance of this site or any other third party hosted site.

## Footnotes

4 The abbreviations used are:

LMP: lysosomal membrane permeabilization
LM: lysosomal membrane
LC: lysosomal content
OSM: oncostatin M
TEM: transmission electron microscopy
ER: endoplasmic reticulum
KEGG: Kyoto Encyclopedia of Genes and Genomes
GO: gene ontology
FDR: false discovery rate
PNGase: peptide-*N*-glycosidase
PNS: post-nuclear supernatant
SN: supernatant
Z: benzyloxycarbonyl
AMC: 7-amino-4-methylcoumarin
emPAI: exponentially modified protein abundance index.
