# Supplementary material for: Stat3-mediated alterations in lysosomal membrane protein composition
Source: J Biol Chem. 2018 Jan 17;293(12):4244–61. doi: 10.1074/jbc.RA118.001777 (PMC5868265; doi:10.1074/jbc.RA118.001777)
Supplement: Supporting Information [file supp_293_12_4244__index.html]

Stat3 mediated alterations in lysosomal membrane protein composition — Stat3 alters proteins in the lysosomal membrane — Stat3-mediated alterations in lysosomal membrane protein composition — Stat3 alters proteins in the lysosomal membrane — Supporting Information 

# Stat3-mediated alterations in lysosomal membrane protein composition

## Supporting Information

- Supporting information - Supporting Figures and Tables
- Supplementary Table S1 - Supplementary Table S1
- Supplementary Table S2 - Supplementary Table S2
- Supplementary Table S3 - Supplementary Table S3
- Supplementary Table S4 - Supplementary Table S4
- Supplementary Table S5 - Supplementary Table S5
- Supplementary Table S6 - Supplementary Table S6
- Supplementary Table S7 - Supplementary Table S7
- Supplementary Table S8 - Supplementary Table S8
- Supplementary Table S9 - Supplementary Table S9
- Supplementary Table S10 - Supplementary Table S10
- Supplementary Table S11 - Supplementary Table S11
- Supplementary Table S12 - Supplementary Table S12
- Supplementary Table S13 - Supplementary Table S13
