# Supplementary material for: Stat3-mediated alterations in lysosomal membrane protein composition
Source: J Biol Chem. 2018 Jan 17;293(12):4244–61. doi: 10.1074/jbc.RA118.001777 (PMC5868265; doi:10.1074/jbc.RA118.001777)
Supplement: Supporting Information [file supp_RA118.001777_135112_0_supp_50079_p24xr9.pdf]

Supporting Information

Stat3 mediated alterations in lysosomal membrane protein composition

Bethan Lloyd-Lewis[1], Caroline C. Krueger[1], Timothy J. Sargeant[2], Michael E. D'Angelo[1], Michael J. Deery[3], Renata Feret[3], Julie A. Howard[3], Kathryn S. Lilley[3] and Christine J. Watson[1*]

[1]Department of Pathology, University of Cambridge, Cambridge, CB2 1QP, UK. [2]Lysosomal Diseases Research Unit, South Australian Health and Medical Research Institute Adelaide, SA, Australia. [3]Cambridge Centre for Proteomics, Department of Biochemistry, University of Cambridge, Tennis Court Road, CB2 1QR, Cambridge, UK.

*Correspondence: Professor Christine Watson

Email: cjw53@cam.ac.uk

Tel: +44 (0)1223 333725

Contents:

Supplementary Figures S1-S4

Supplementary Tables (.xls files):

Supplementary Table S1A and S1B: Mass spec raw data from pilot experiment.

Supplementary Table S2: Proteins identified from pilot lysosomal MS experiment - excluding hits identified in unlabelled control sample and common contaminants

Supplementary Table S3A and S3B. Kyoto Encyclopedia of Genes and Genomes (KEGG) pathway analysis and Cellular Component (CC) GO term enrichment analysis of the proteins identified in the pilot experiment

Supplementary Table S4: Mass spec raw data for 3 biological replicates of EpH4 lysosomal preparations treated with or without OSM

Supplementary Table S5: MS samples table for 3 biological replicates of EpH4 lysosomal preparations

Supplementary Table S6: Exclusive unique peptide counts for all proteins identified in vehicle and OSM treated EpH4 cell lysosomes

Supplementary Table S7: Proteins identified in vehicle and OSM treated EpH4 cell lysosomes - excluding hits identified in unlabelled control sample and common contaminants

Supplementary Table S8: List of 320 (out of 447) proteins that were identified in 5 out of 6 lysosomal preparations

Supplementary Table S9: Kyoto Encyclopedia of Genes and Genomes (KEGG) pathway analysis and Cellular Component (CC) GO term enrichment analysis of the 320 proteins identified in 5 out of 6 samples (associated with Figure 4)

Supplementary Table S10: Exclusive unique peptide counts of 39 proteins whose representation changed with OSM stimulation

Supplementary Table S11: Microarray data from Stat3 knockout and control mammary glands at 24h involution showing fold downregulation of *Flot1, Flot2* and *CD63* mRNA in Stat3 knockout samples.

Supplementary Table S12: Raw MS data from deglycosylation experiment

Supplementary Table S13: Representation of heavily glycosylated lysosomal membrane proteins with or without PNGase F enzyme treatment

Supplementary Figure S1

A

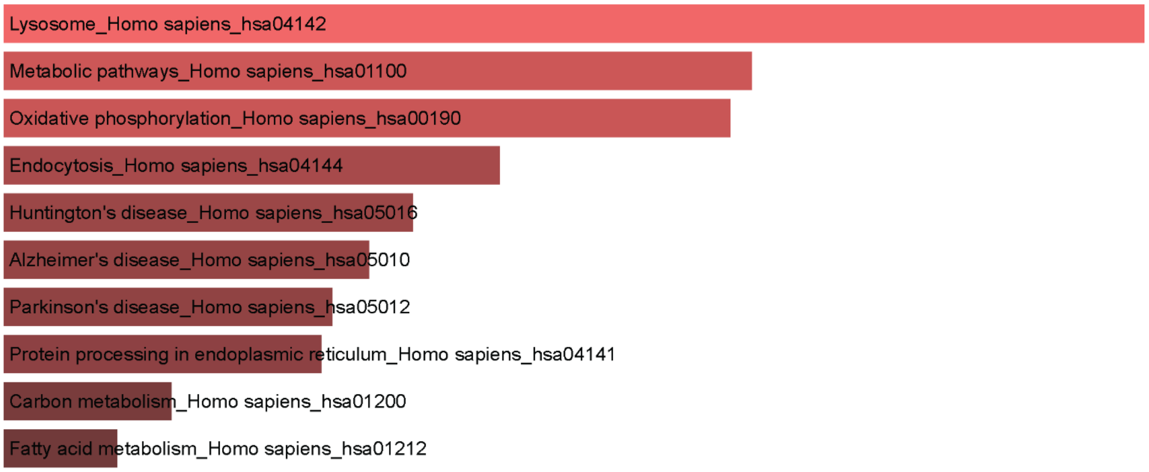

B

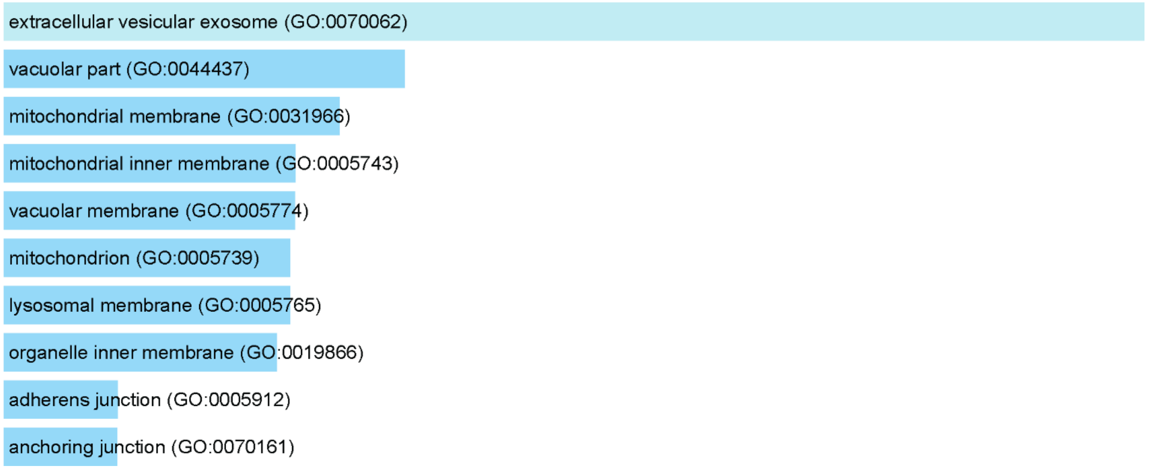

Supplementary Figure S1.

A, Significantly enriched KEGG (2016) pathways derived from mass-spectrometry analysis of EpH4 lysosomal preparations isolated using iron nanoparticles. **B**, Significantly enriched GO annotations according to the category Cellular Component. Associated with Supplementary Table S3.

Supplementary Figure S2

A

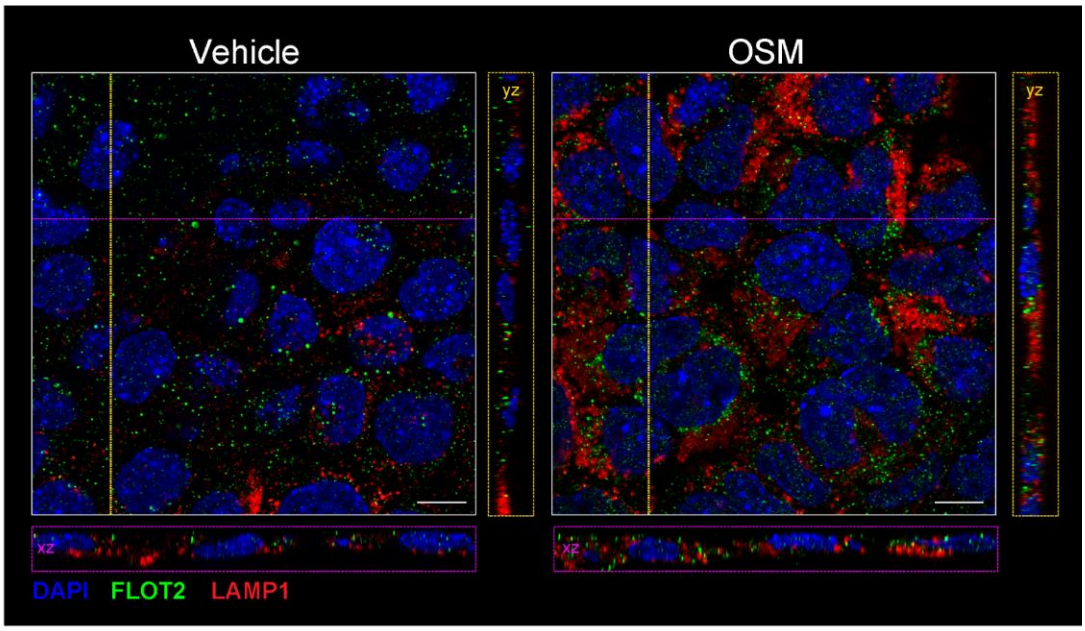

B

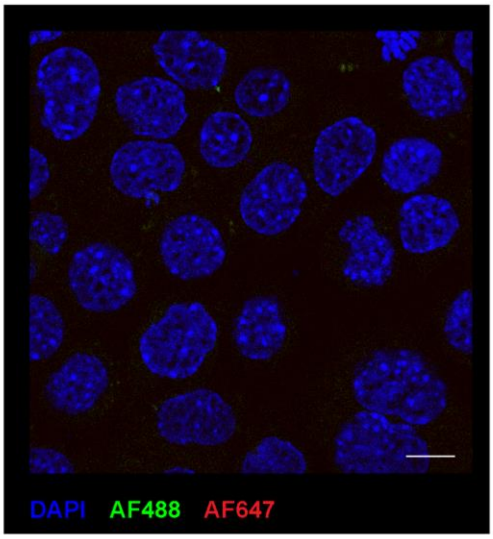

Supplementary Figure S2.

A, Confocal fluorescence imaging of EpH4 cells treated with OSM for 24 h and immunostained for flotillin 2 and LAMP1. Main images show a single thin optical slice, orthagonal views show *XZ* (purple line and box) and *YZ* (orange line and box) planes. Scale bars: 10 µm. Representative images of n=2 independent experiments are shown. **B,** No primary antibody controls. Scale bar: 10 µm

Supplementary Figure S3

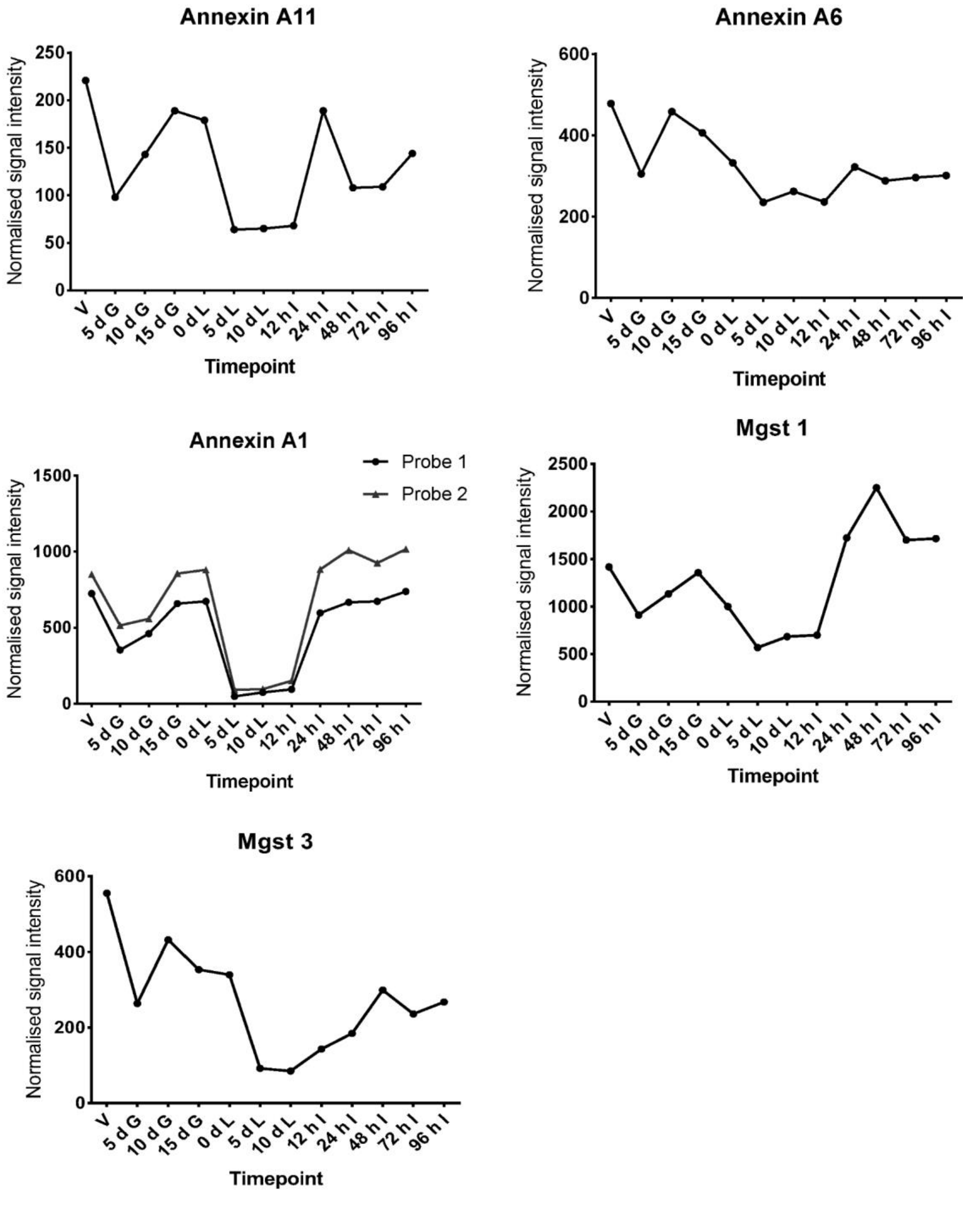

Supplementary Figure S3.

Microarray analysis of twelve different timepoints during the mammary gland pregnancy cycle showing the involution related expression profiles of selected proteins that were more abundant in OSM treated EpH4 cell lysosomes. V, virgin; d G, days gestation; d L, days lactation; h I, hours involution.

Supplementary Figure S4

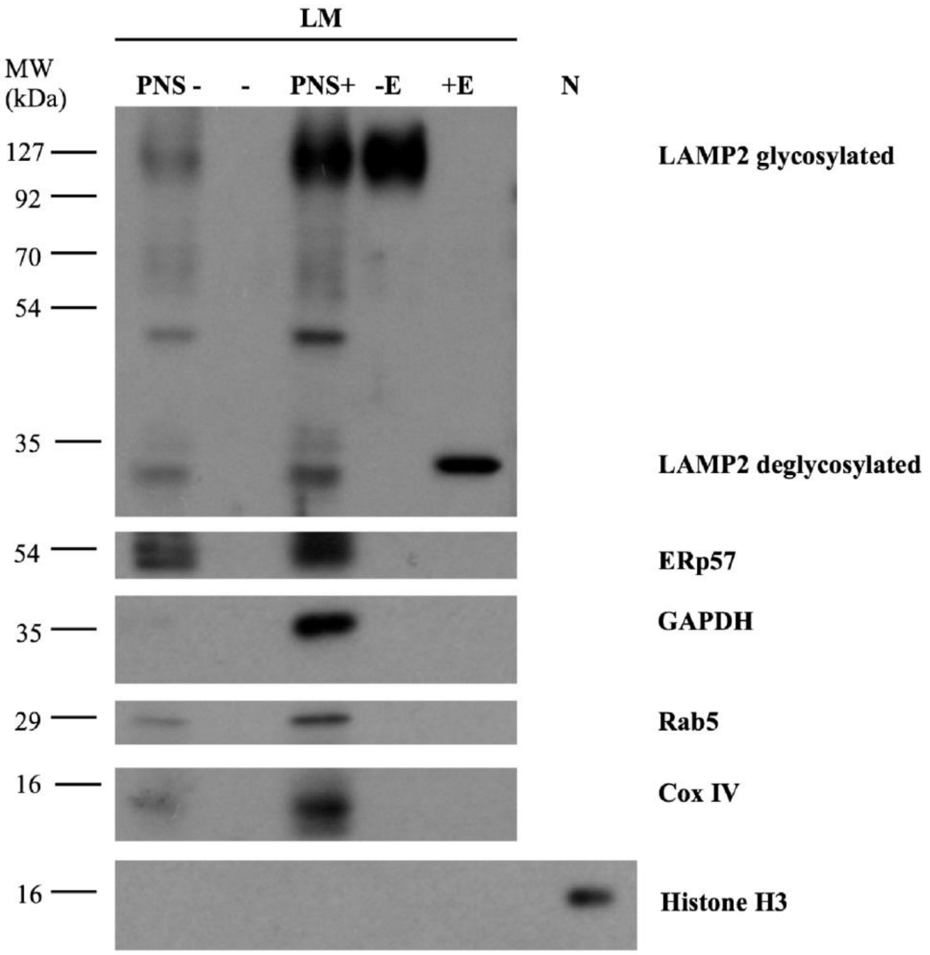

Supplementary Figure S4.

LAMP2 deglycosylation by PNGase F. EpH4 cell lysosomes were isolated using the magnetic nanoparticle separation protocol and fractionated by freezing in liquid nitrogen and thawing at 37°C five times. The lysosomal membrane fraction (LM) was either untreated (UT) or denaturated at 95°C and incubated with (+ E) or without (- E) PNGase F for 30 min at 37°C. N, nuclear lysate. Organelle marker proteins: LAMP2, lysosomes; ERp57, endoplasmic reticulum; GAPDH, cytoplasm; Rab5, early endosomes; Cox IV, mitochondria; Histone H3, nucleus.
